# Designed whole-cell-catalysis-assisted synthesis of 9,11-secosterols

**DOI:** 10.3762/bjoc.17.52

**Published:** 2021-03-01

**Authors:** Marek Kõllo, Marje Kasari, Villu Kasari, Tõnis Pehk, Ivar Järving, Margus Lopp, Arvi Jõers, Tõnis Kanger

**Affiliations:** 1 Department of Chemistry and Biotechnology, School of Science, Tallinn University of Technology, Akadeemia tee 15, 12618 Tallinn, Estonia; 2 Institute of Technology, University of Tartu, Nooruse 1, 50104 Tartu, Estonia; 3 National Institute of Chemical Physics and Biophysics, Akadeemia tee 23, 12618 Tallinn, Estonia

**Keywords:** chemoenzymatic synthesis, cortisol, hydroxylation, secosterol, whole-cell catalysis

## Abstract

A method for the synthesis of 9,11-secosteroids starting from the natural corticosteroid cortisol is described. There are two key steps in this approach, combining chemistry and synthetic biology. Stereo- and regioselective hydroxylation at C9 (steroid numbering) is carried out using whole-cell biocatalysis, followed by the chemical cleavage of the C–C bond of the vicinal diol. The two-step method features mild reaction conditions and completely excludes the use of toxic oxidants.

## Introduction

**Figure 1:**
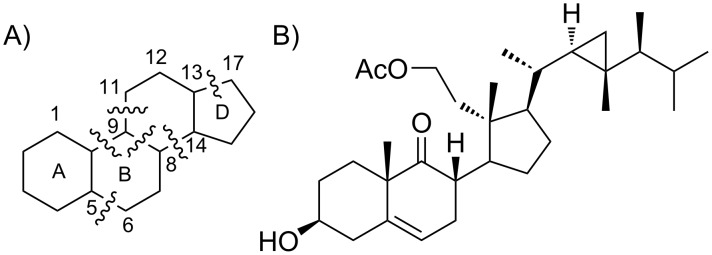
A) Tetracyclic core of steroids and possible sites of bond cleavages for secosteroids. B)The first 9,11-secosteroid isolated in 1972 [7].

Developments in the chemistry of steroids have stimulated extensive research interest in the exploration of new synthetic methods since the 1960s. Advances in synthetic biology and the increasing importance of methods of sustainable chemistry have brought chemoenzymatic approaches for obtaining natural products, or derivatives thereof, with complex structure into focus [1]. Various enzymatic or semisynthetic methods have also been exploited in the synthesis of steroids [2–4]. A more challenging synthesis of secosteroids with unique broken tetracyclic carbon skeletons and abeo-steroids with migrated bonds has received attention only recently [5–6]. As the bond cleavage may occur at the 5,6-, 9,11-, 9,10-, 8,9-, 8,14- or 13,17-positions (Figure 1A), the selection of synthetic methods for secosteroids is wide. However, usually these are multistep sequences exploiting toxic oxidants.

Marine invertebrates are a rich source of oxidated and highly functionalized steroidal metabolites, including secosteroids. Since the first isolation of 9,11-secosterol from *Pseudopterogorgia americana* in 1972 (Figure 1B) [7], several others from the family have been reported [8–14]. The 9,11-secosterols exhibit diverse biological activities, including antihistaminic, antiproliferative, anti-inflammatory, cytotoxic and protein kinase C (PKC) inhibition activities [8–10,15]. Biochemical characterization of 9,11-secosterols has so far mainly relied on the identification and purification of natural products from marine invertebrates. The intriguing profile of biological properties has prompted synthetic studies of this class of secosterols. The majority of synthetic schemes starts with natural steroids, taking advantage of the appropriate stereochemistry of existing stereogenic centres [16–20]. However, the synthesis of target compounds is a multistep procedure, often including several protections and deprotections of functional groups.

**Scheme 1:**
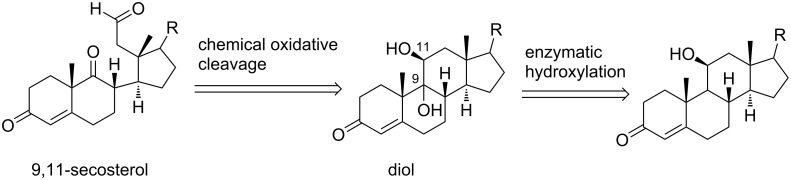
Retrosynthetic analysis of 9,11-secosterols.

Our approach to 9,11-secosterols is depicted in the retrosynthetic analysis in Scheme 1. There are two key steps in obtaining the skeleton of the secosterol. The first is (di)hydroxylation at C9 (C11), and the second is C9–C11-bond cleavage, which can be carried out by a well-developed chemical oxidation of 1,2-diols. Starting with a compound already possessing a hydroxy group at the position C11, only hydroxylation at C9 is needed. Commercially available corticosteroid cortisol already possesses a hydroxy group at the position C11, and therefore only hydroxylation at C9 is needed, making cortisol (**1**) an ideal starting compound for this synthesis.

Chemical oxidation methods for the synthesis of steroids often require stoichiometric amounts of toxic reagents, and the selectivity of the oxidation is still an unsolved problem [21].

Typically, toxic oxidants, such as OsO_4_, SeO_2_ and Pb(OAc)_4_ are used in this total synthesis sequence. Inspired by several enzymatic oxidations [22–25], we envisioned to carry out oxidation at C9 in an environmentally benign way using an oxidation by a whole-cell biocatalysis method.

The first, and so far the only chemoenzymatic synthesis of 9,11-secosterols using cellular lysate of the marine gorgonian *Pseudopterogorgia americana* was published by Keliman et al. in 1996 [26]. They carried out quite effective transformations of a variety of sterols to 9,11-secosteroid derivatives in high yields. However, it is unclear which enzymes were responsible for each oxidation step through the whole secosteroid synthesis pathway. Also, the use of cellular lysate from a nonlaboratory organism prevents any widespread adoption of this method.

Herein, we present a new combined route towards 9,11-secosterols via stereo- and regiospecific enzymatic hydroxylation at C9, followed by the chemical oxidative cleavage of the 9,11-C–C bond. For the hydroxylation, we used a biocatalyst derived from an *Escherichia coli* laboratory strain BL21 (DE3) overexpressing the *kshA5* and *kshB* genes from *Rhodococcus. rhodochrous*. Cortisol (**1**) was chosen as a model steroidal structure.

## Results and Discussion

### Synthesis of starting compounds for enzymatic hydroxylation

**Scheme 2:**
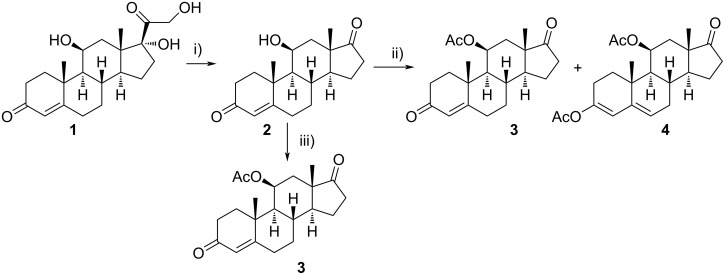
Synthesis of starting materials. Reagents and conditions: i) NaBH_4_, EtOH/CH_2_Cl_2_ 1:1, 2 h, rt, then acetone, H_2_O, NaIO_4_, overnight, rt, 99%; ii) Ac_2_O, *p*-TsOH (1 mol %), MW (800 W), 6 min, 52% for **3** and 33% for **4**; iii) Ac_2_O, DMAP, Et_3_N, CH_2_Cl_2_, overnight, rt, 93%.

In order to estimate the possible diversity of the substrates as starting materials for the biocatalytic transformation, cortisol (**1**) was converted to a hydroxylated steroid derivative **2** by reduction of the C20 carbonyl group with NaBH_4_ and subsequent oxidative cleavage of the intermediate 17,20,21-trihydroxy side chain with NaIO_4_ in 99% total yield (Scheme 2) [27].

The C11 hydroxy group was protected with acetic anhydride in the presence of a base, resulting in C11-protected product **3** in 93% yield. Performing the same reaction under microwave irradiation, a mixture of monoacylated and diacylated products **3** and **4** in 52% and 33% yield, respectively, was isolated [28].

### Enzymatic hydroxylation

3-Ketosteroid 9α-hydroxylase (KSH) from *R. rhodochrous* has been shown to oxidize C9 in several steroids [29]. This enzyme consists of two polypeptides: KshA (terminal oxygenase) and KshB (ferredoxin reductase). When expressed together in *E. coli*, active KSH is formed, and several steroids can be oxidized in the C9 position [29–30]. Out of five KshA homologues found in *R. rhodochrous*, only KshA5 is able to utilize C11-hydroxylated cortisone, 11β-hydrocortisone, as a substrate [30].

For the construction of the biocatalyst, *kshA5* and *kshB* genes from *R. rhodochrous* were codon-optimized for enhanced expression in *E. coli* and cloned into a pET21a protein expression plasmid. To obtain an active biocatalyst, the plasmid was transformed into an *E. coli* BL21 (DE3) strain, and protein expression was induced by the addition of IPTG. Substrates of biocatalysis were added together with the IPTG inducer. The whole-cell biocatalysis was performed overnight at 30 °C in a rich medium with continuous shaking. Cell pellet and culture supernatant were collected the next morning for further analysis. A typical reaction contained approximately 40 mg of substrate in 200 mL culture medium, depending on the solubility and availability of substrates. This reaction can be scaled up by increasing the culture volume; the substrate concentration cannot be increased due to the low solubility of corticosteroids in aqueous solution. The expression of KshA5 and KshB was verified from the cell lysate by polyacrylamide gel electrophoresis and western blotting (see Supporting Information File 1, Figure S1). The product distribution between the cellular pellet and supernatant was approximately 1:10 (estimated by HRMS analysis). The steroid compounds were extracted from the supernatant and purified by column chromatography on silica gel as the stationary phase. The outcome of the enzymatic hydroxylation in position C9 using KSH-based biocatalysis is given in Table 1.

**Table 1:** Enzymatic hydroxylation.

entry	substrate	product	yield (%)^a^

1	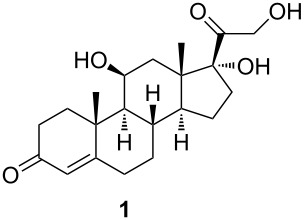	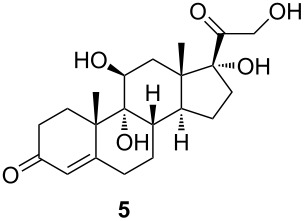	29
2	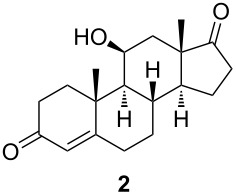	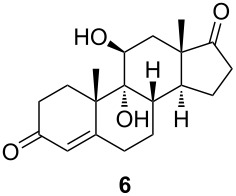	27
3^b^	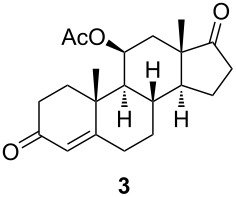	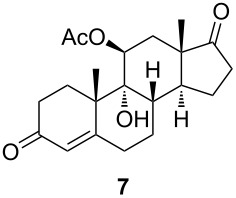	9
4^c^	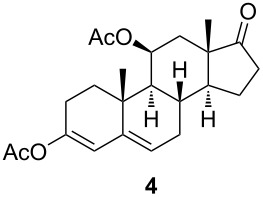	–	

^a^Isolated yield after column chromatography. ^b^23% of starting material was recovered. ^c^No products were formed, only starting substrate was detected.

With substrates **1** and **2**, enzymatic hydroxylation proceeded efficiently and selectively at C9, affording *trans*-9,11-dihydroxysteroids **5** and **6** , respectively. For compound **5**, 67% of the starting compound was isolated. In Table 1, entry 2, several unidentified products were also formed. With 11-acetoxy steroid **3**, the C9-hydroxylated product **7** was isolated together with unreacted starting steroid **2**. Conjugated enol ester **4** did not react, and only the starting compound was detected in the reaction mixture. This indicates that the C3 carbonyl group in substrates is essential for enzymatic hydroxylation, as has been shown before [29]. A positive control experiment with the same batch of KSH-expressing *E. coli* cells using cortisol (**1**) as a substrate yielded C9-hydroxylated product **5**. From the results, we can also conclude that a protected C11 hydroxy group decreases the effectiveness of enzymatic transformation. Also, the substituent at C17 does not affect the KSH performance: steroids with C17 keto functionality (i.e., **2** and **3**) or cholesterol-like side chains at C20 (i.e., **1**) were all transformed efficiently to the corresponding 9,11-dihydroxylated products. ^1^H and ^13^C 1D and 2D (COSY, HSQ and HMBC) NMR spectra from samples **5** and **6** were analyzed, as were the spectra of the starting compounds **1** and **2**. The assignment of all ^1^H and ^13^C signals confirmed the preservation of the configuration of the ring system and substituents in these whole-cell transformations, as well as the OH group connections to C9.

### Chemical oxidation of 9,11-dihydroxysteroids

**Scheme 3:**
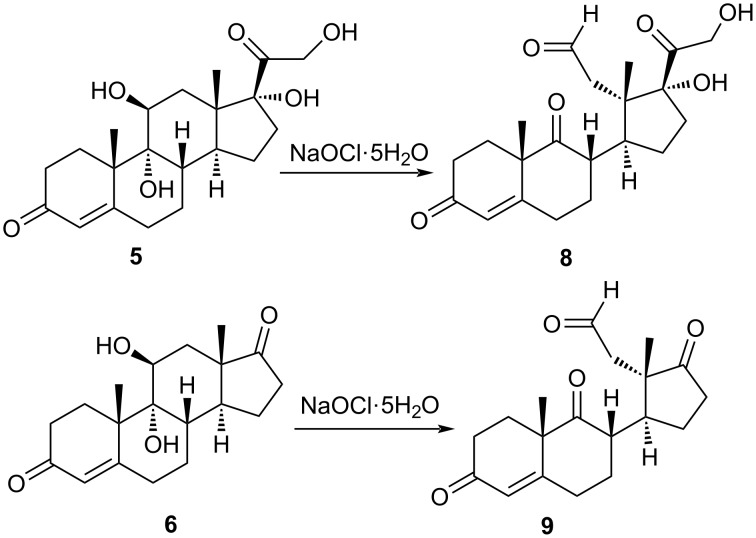
Oxidation of diols **5** and **6** with NaOCl·5H_2_O.

In the final step, the obtained *trans*-diols **5** and **6** were subjected to the chemical oxidation of the 9,11 carbon bond. Lead tetraacetate is a classic oxidant for the cleavage of vicinal diols. However, there are serious drawbacks when using Pb(OAc)_4_. In addition to its toxicity, the oxidation rate affording *trans*-diols was very slow in comparison to that for *cis*-diols. Using an even longer reaction time and stoichiometric amount of the reagent, diol **5** was not oxidized to the corresponding dicarbonyl compound **8**. However, using NaOCl·5H_2_O as an oxidant [31], diols **5** and **6** were effectively converted to the corresponding 9,11-secosterols **8** and **9** within one hour at 0 °C (Scheme 3).

Compound **8** was isolated as 2:1 mixture together with starting diol **5** and purified further by preparative TLC. Compound **9** was isolated as a single product in 87% yield.

## Conclusion

We have presented the results of the synthesis of the 9,11-secosteroid carbon skeleton by using the designed whole-cell biotransformation of natural steroids with a genetically engineered biocatalyst. The enzymatic oxidation of cortisol derivatives is completely stereo- and regioselective, affording only 9α-hydroxylated diol. The following oxidative cleavage of the C–C bond with a mild oxidant leads to the steroid with an appropriately broken steroid skeleton. The method provides the target compound in only two steps, without any manipulations involving protecting groups. The present method features mild reaction conditions, and the protocol tolerates several functional groups. It is an environmentally benign approach, totally excluding the use of highly toxic oxidants. Our synthetic scheme provides a direct entry to structurally diverse 9,11-secosterols, enabling also studies of their biological properties. Further research to broaden the scope of the presented synthetic scheme is ongoing.

## Experimental

### General data

Full assignment of ^1^H and ^13^C chemical shifts were based on the 1D and 2D FT NMR spectra, measured with a Bruker Avance III 400 MHz or a Bruker Avance III 800 MHz instrument. Residual solvent signals were used (CDCl_3_: δ = 7.26 for ^1^H NMR, δ = 77.2 for ^13^C NMR or CD_3_OD: δ = 3.31 for ^1^H NMR, δ = 49.0 for ^13^C NMR) as internal standards. Optical rotations were obtained by using an Anton Paar GWB Polarimeter MCP500. High-resolution mass spectra were recorded with an Agilent Technologies 6540 UHD Accurate-Mass QTOF LC/MS spectrometer by using AJ-ESI as an ionization method.

### Construction of biocatalyst

Plasmid cloning and amplification were performed in *E. coli* DH5 strain. *E. coli* strain BL21 (DE3) was used for biocatalysis experiments. Lysogeny broth (LB) medium was used for all cell-growth incubations. For the selection of plasmids, 100 μg/mL ampicillin was used.

Plasmid pAJ30 for heterologous expression of *kshA5* and *kshB* was constructed using the CPEC method [32]. *kshA5* and *kshB* gene sequences from *R. rhodochrous* were codon-optimized for high-level expression in *E. coli* using the IDT codon optimization tool (to eliminate rare codons). Synthetic DNA was ordered from Twist Bioscience and inserted into pET21a backbone as one operon (see Supporting Information File 2) under the control of an IPTG-inducible tac promoter. The obtained plasmid was verified by DNA sequencing. To obtain the biocatalyst strain, the plasmid pAJ30 was transformed into chemically competent *E. coli* BL21 (DE3) cells.

### Enzymatic hydroxylation

3 mL precultures of BL21(DE3) transformed with pAJ30 plasmid (biocatalyst) or without plasmid (negative control) were grown overnight at 37 °C. The next morning, 200 mL of LB medium in a 2 L baffled flask was inoculated with 1 mL of a preculture. The cultures were grown until OD_600_ = 0.1 at 37 °C with continuous shaking at 220 rpm, and then the expression was induced by adding 1 mM IPTG and a substrate (compound **1**–**4**, respectively) at a final concentration of 225 μg/mL, 175 μg/mL, 190 μg/mL or 122 μg/mL, respectively. The biocatalysis was carried out for 20 hours at 30 °C with continuous shaking at 220 rpm. The next morning, the cells were harvested by centrifugation at 4,000*g*, and cell pellet and culture supernatant were stored at a temperature of −20 °C until further analysis.

### Synthesis of substrates

#### 11β-Hydroxyandrost-4-ene-3,17-dione (**2**)

To a stirred solution of cortisol (**1**, 1.2 g, 3.36 mmol) in a 1:1 mixture of EtOH and CH_2_Cl_2_ (23 mL), NaBH_4_ (51.2 mg, 1.35 mmol) was added in one portion at room temperature. After 2 h, acetone (5.8 mL) was added, followed by water (5.8 mL) and NaIO_4_ (1.8 g, 8.41 mmol). The solution was stirred overnight at room temperature. Water (115 mL) was added, and the reaction mixture was extracted with CHCl_3_ (3 × 50 mL). The combined organic layers were dried over sodium sulfate and filtered. After evaporation of the solvent, ketone **2** (1.01 g, 99%) was isolated as white amorphous solid. [α]_D_^20^ +177.4 (*c* 0.46, CHCl_3_); ^1^H NMR (CDCl_3_, 400 MHz) δ 5.69 (d, *J =* 1.3 Hz, 1H), 4.45 (q, *J =* 3.0 Hz, 1H), 2.58–1.97 (m, 10H), 1.95 (dd, *J =* 14.4, 2.7 Hz, 1H), 1.85 (td, *J =* 13.5, 4.6 Hz, 1H), 1.73–1.58 (m, 1H), 1.52–1.47 (m, 1H), 1.46 (s, 3H), 1.34–1.18 (m, 2H), 1.16 (s, 3H), 1.14–1.06 (m, 1H), 1.00 (dd, *J =* 11.1, 3.3 Hz, 1H); ^13^C NMR (CDCl_3_, 101 MHz) δ 219.19, 199.48, 171.63, 122.70, 68.07, 56.80, 52.49, 46.84, 41.13, 39.43, 35.41, 35.15, 33.93, 31.95, 31.62, 31.09, 21.80, 21.20, 15.99; HRMS (*m*/*z*): [M + H]^+^ calcd, 303.1955; found, 303.1961.

#### 11β-Acetoxyandrost-4-ene-3,17-dione (**3**)

Starting steroid **2** (202 mg, 0.67 mmol, 1 equiv) was dissolved in dry dichloromethane (9.8 mL), and after that 4-(dimethylamino)pyridine (8.2 mg, 0.067 mmol, 0.1 equiv), triethylamine (373 μL, 2.67 mmol, 4 equiv) and acetic anhydride (316 μL, 3.34 mmol, 5 equiv) were added. The mixture was stirred under argon atmosphere overnight at room temperature. Saturated aqueous NH_4_Cl (4.11 mL) was added, and the reaction mixture was extracted with CH_2_Cl_2_ (4 × 6 mL). The combined organic layers were dried over sodium sulfate, filtered and then concentrated in vacuo. The residue was chromatographed on silica gel with gradient 10% to 40% acetone in petroleum ether to give acetate **3** (213.2 mg, 93%) as pale yellow oil. [α]_D_^20^ +180.0 (*c* 0.58, CHCl_3_); ^1^H NMR (CDCl_3_, 400 MHz) δ 5.68 (d, *J =* 1.4 Hz, 1H), 5.47 (q, *J =* 3.1 Hz, 1H), 2.57–2.21 (m, 6H), 2.20–2.05 (m, 4H), 2.04 (s, 3H), 1.99 (m, 1H), 1.85–1.74 (m, 1H), 1.69–1.60 (m, 1H), 1.42 (dd, *J =* 14.9, 3.3 Hz, 1H), 1.28 (s, 3H), 1.21–1.10 (m, 3H), 1.03 (s, 3H); ^13^C NMR (CDCl_3_, 101 MHz) δ 217.94, 198.85, 170.13, 169.58, 123.08, 69.41, 55.60, 52.11, 46.48, 38.76, 36.99, 35.48, 35.27, 33.71, 31.82, 31.65, 31.34, 21.90, 21.71, 20.87, 15.56; HRMS (*m*/*z*): [M + H]^+^ calcd, 345.2060; found, 345.2078.

#### 3,11β-Bis(acetyloxy)androsta-3,5-dien-17-one (**4**)

The general procedure of Marwah et al. [28] was followed with modifications. A mixture of steroid **2** (254 mg, 0.84 mmol, 1 equiv), acetic anhydride (475 μL, 5.03 mmol, 6 equiv) and *p*-toluenesulfonic acid monohydrate (2.8 mg, 0,008 mmol, 0,01 equiv) in a beaker was subjected to continuous mode of microwave irradiation (800 W) at high power setting in a domestic microwave oven for 6 min. After this, the mixture was cooled to room temperature, and saturated aqueous NaHCO_3_ (5 mL) was added, after which this was mixed and extracted with EtOAc (5 mL). The organic layer was dried over magnesium sulfate, filtered and concentrated in vacuo. The residue was chromatographed on silica gel with gradient 7% to 30% acetone in petroleum ether to give monoacetate **3** (151 mg, 52%) and diacetate **4** (107 mg, 33%). Diacetate **4**: ^1^H NMR (CDCl_3_, 400 MHz) δ 5.66 (d, *J =* 2.1 Hz, 1H), 5.50 (q, *J =* 3.2 Hz, 1H), 5.33 (t, *J =* 3.8 Hz, 1H), 2.67–2.36 (m, 4H), 2.29 (m, 1H), 2.12 (s, 3H), 2.11–2.04 (m, 4H), 2.03 (s, 3H), 2.01–1.96 (m, 1H), 1.95–1.84 (m, 1H), 1.75 (dd, *J =* 12.7, 4.2 Hz, 1H), 1.70–1.62 (m, 1H), 1.51–1.12 (m, 2H), 1.08 (s, 3H), 1.03 (s, 3H); ^13^C NMR (CDCl_3_, 101 MHz) δ 218.48, 169.85, 169.41, 147.42, 140.45, 122.24, 116.12, 69.66, 53.31, 50.99, 46.72, 37.01, 35.38, 34.90, 33.49, 30.73, 28.37, 24.60, 21.96, 21.79, 21.54, 21.21, 15.50; HRMS (*m*/*z*): [M + H]^+^ calcd, 387.2166; found, 387.2167.

### Isolation of 9-hydroxysteroids

A frozen culture supernatant was melted at room temperature or in a warm water bath, then poured into the separation funnel, saturated by the addition of solid sodium chloride and extracted with EtOAc. The extract was concentrated in vacuo to give a solid residue that was chromatographed on silica gel. Elution with acetone/petroleum ether solvent system (30% to 50% for **5** and **6**, 10% to 30% for **7**) afforded the product.

### 9-Hydroxysteroids

#### 9α,11β,17α,21-Tetrahydroxypregn-4-ene-3,20-dione (**5**)

Compound **5** was further purified by column chromatography on silica gel with 10% MeOH/CHCl_3_. [α]_D_^20^ +119.2 (*c* 0.66, MeOH); ^1^H NMR (MeOD, 400 MHz) δ 5.72 (s, 1H), 4.64 (d, *J =* 19.2 Hz, 1H), 4.26 (d, *J =* 19.1 Hz, 1H), 4.05 (t, 1H), 2.73 (ddd, *J =* 14.2, 11.2, 2.7 Hz, 1H), 2.67–2.54 (m, 1H), 2.53–2.46 (m, 2H), 2.43–2.22 (m, 8H), 2.02–1.94 (m, 1H), 1.70 (dt, *J =* 10.9, 5.6 Hz, 2H), 1.58 (s, 3H), 1.49 (ddd, *J =* 14.9, 9.2, 6.4 Hz, 1H), 1.42–1.36 (m, 2H), 0.87 (s, 3H); ^13^C NMR (CD_3_OD, 201 MHz) δ 213.08, 202.81, 176.40, 124.85, 90.50, 78.81, 73.87, 67.69, 48.27, 46.75, 46.25, 36.83, 35.36, 34.86, 34.75, 32.33, 29.11, 26.84, 24.54, 22.49, 17.60; HRMS (*m*/*z*): [M + H]^+^ calcd, 303.1955; found, 303.1961.

#### 9α,11β-Dihydroxyandrost-4-ene-3,17-dione (**6**)

^1^H NMR (CD_3_OD, 800 MHz) δ 5.74 (ddd, *J =* 2.1, 0.8, 0.5 Hz, 1H), 4.03 (dd, *J =* 3.3, 2.6 Hz, 1H), 2.64 (dddd, *J =* 15.4, 13.3, 6.9, 2.1 Hz, 1H), 2.53 (ddd, *J =* 12.5, 11.2, 4.7 Hz, 1H), 2.52 (ddd, *J =* 16.1, 14.4, 5.3 Hz, 1H), 2.48 (ddd, 19.2, 8.8, 0.9 Hz, 1H), 2.45 (dddd, *J =* 14.4, 13.0, 4.3, 0.5 Hz, 1H), 2.34 (dddd, *J =* 16.1, 4.3, 3.1, 0.8 Hz, 1H), 2.30 (dddd, *J =* 15.4, 5.6, 1.5, 0.5 Hz, 1H), 2.06 (dt, *J =* 19.2, 8.8, 8.8 Hz, 1H), 1.99 (ddd, *J =* 13.0, 5.3, 3.2 Hz, 1H), 1.91 (m, 1H), 1.89 (m, 1H), 1.82 (ddddd, *J =* 13.3, 6.9, 4.7, 1.5, 0.5 Hz, 1H), 1.73 (bdd, *J =* 14.0, 3.3 Hz, 1H), 1.72 (dd, *J =* 14.0, 2.6 Hz, 1H), 1.68 (m, 1H), 1.66 (dddd, *J =* 13.3, 13.3, 12.5, 5.6 Hz, 1H), 1.61 (d, *J =* 0.5 Hz, 3H), 1.16 (s, 3H); ^13^C NMR (CD_3_OD, 201 MHz) δ 222.73, 202.58, 175.91, 124.93, 79.13, 73.30, 48.17, 46.78, 46.50, 37.32, 36.34, 34.80, 34.76, 32.08, 29.01, 25.55, 22.46, 22.35, 15.89; HRMS (*m*/*z*): [M + H]^+^ calcd, 319.1904; found, 319.1866.

#### 9α-Hydroxy-11β-acetyloxyandrost-4-ene-3,17-dione (**7**)

[α]_D_^20^ +157.3 (*c* 0.23, CHCl_3_); ^1^H NMR (MeOD, 400 MHz) δ 5.75 (d, *J =* 1.9 Hz, 1H), 5.10 (t, *J =* 3.0 Hz, 1H), 2.60–2.41 (m, 4H), 2.38–2.27 (m, 2H), 2.16 (d, *J =* 9.5 Hz, 4H), 2.07 (s, 3H), 2.05–1.91 (m, 1H), 1.85 (dddd, *J =* 13.4, 6.8, 4.8, 1.7 Hz, 1H), 1.78 (d, *J =* 3.0 Hz, 2H), 1.73–1.64 (m, 1H), 1.62–1.52 (m, 1H), 1.49 (s, 3H), 1.05 (s, 3H); ^13^C NMR (MeOD, 101 MHz) δ 221.00, 201.81, 174.03, 170.77, 125.22, 56.05, 47.45, 46.22, 46.01, 36.17, 35.43, 34.63, 33.93, 32.02, 29.74, 29.53, 25.38, 22.82, 22.25, 21.63, 15.59; HRMS (*m*/*z*): [M + Na]^+^ calcd, 383.1829; found, 383.1825.

### Chemical oxidation of 9,11-dihydroxysteroids

The general procedure of Kirihara et al. [31] was followed. Sodium hypochlorite pentahydrate (3 equiv) was added to a stirred solution of diol (1 equiv) and tetrabutylammonium hydrogen sulfate (0.1 equiv) in dichloromethane (8 mL/mmol diol) and water (2.7 mL/mmol diol) at 0 °C. The resulting mixture was stirred for 1 h and monitored by TLC analysis (10% MeOH in CHCl_3_). Water (2.7 mL/mmol diol) was added, and the reaction mixture was extracted with CH_2_Cl_2_ (2 × 32 mL/mmol diol). The combined organic layers were dried over anhydrous sodium sulfate, filtered and then concentrated in vacuo.

#### 17α,21-Dihydroxy-3,9,20-trioxo-9,11-seco-pregn-4-en-11-al (**8**)

Compound **8** was obtained as a mixture with **5** in the ratio 2:1. Compound **8** was further purified by preparative TLC (10% MeOH in CHCl_3_); ^1^H NMR (MeOD, 400 MHz) δ 9.60 (s, 1H, H-11), 5.81 (d, *J =* 2.3 Hz, 1H, H-4), 1.50 (s, 3H, 19-Me), 0.83 (s, 3H, 18-Me); ^13^C NMR (CDCl_3_, 101 MHz) δ 221.31 (C9), 211.52 (C20), 200.50 (C11), 198.19 (C3), 165.72 (C5), 125.90 (C4); HRMS (*m*/*z*): [M + H]^+^ calcd, 377.1959; found, 377.1944.

#### 3,9-Dioxo-9,11-secoandrost-4-en-11-al (**9**)

Compound **9** was obtained as pure compound with a yield of 87%. [α]_D_^20^ +57.1 (*c* 0.42, CHCl_3_); ^1^H NMR (CDCl_3_, 400 MHz) δ 9.69 (d, *J =* 0.8 Hz, 1H), 5.83 (d, *J =* 2.0 Hz, 1H), 3.40–3.28 (m, 1H), 3.00–2.73 (m, 5H), 2.66–2.54 (m, 2H), 2.51–2.36 (m, 2H), 2.30–2.16 (m, 1H), 2.10–2.01 (m, 1H), 1.95 (ddd, *J =* 14.1, 4.8, 3.2 Hz, 1H), 1.74–1.53 (m, 1H), 1.51 (s, 3H), 1.49–1.37 (m, 2H), 0.84 (s, 3H); ^13^C NMR (CDCl_3_, 101 MHz) δ 221.30, 211.50, 200.48, 198.17, 165.72, 125.87, 51.55, 51.30, 48.56, 47.37, 39.82, 36.20, 33.60, 32.45, 29.62, 28.98, 23.72, 22.99, 18.49; HRMS (*m*/*z*): [M + H]^+^ calcd, 317.1747; found, 317.1755.

## Supporting Information

File 1 General material and methods for the construction of the biocatalyst as well as NMR spectra of synthesized compounds.

File 2 DNA sequence.

## References

[1] Li J, Amatuni A, Renata H (2020). Curr Opin Chem Biol.

[2] Fryszkowska A, Peterson J, Davies N L, Dewar C, Evans G, Bycroft M, Triggs N, Fleming T, Gorantla S S C, Hoge G (2016). Org Process Res Dev.

[3] Carvalho J F S, Silva M M C, Moreira J N, Simões S, Melo M L S (2009). J Med Chem.

[4] Contente M L, Molinari F, Serra I, Pinto A, Romano D (2016). Eur J Org Chem.

[5] Noack F, Heinze R C, Heretsch P (2019). Synthesis.

[6] Duecker F L, Reuß F, Heretsch P (2019). Org Biomol Chem.

[7] Enwall E L, van der Helm D, Hsu I N, Pattabhiraman T, Schmitz F J, Spraggins R L, Weinheimer A J (1972). J Chem Soc, Chem Commun.

[8] Koljak R, Pehk T, Järving I, Liiv M, Lopp A, Varvas K, Vahemets A, Lille Ü, Samel N (1993). Tetrahedron Lett.

[9] Lopp A, Pihlak A, Paves H, Samuel K, Koljak R, Samel N (1994). Steroids.

[10] Koljak R, Lopp A, Pehk T, Varvas K, Müürisepp A-M, Järving I, Samel N (1998). Tetrahedron.

[11] Huang C-Y, Su J-H, Duh C-Y, Chen B-W, Wen Z-H, Kuo Y-H, Sheu J-H (2012). Bioorg Med Chem Lett.

[12] Chang Y-C, Hwang T-L, Kuo L-M, Sung P-J (2017). Mar Drugs.

[13] He Y-Q, Lee Caplan S, Scesa P, West L M (2017). Steroids.

[14] Chang Y-C, Lai K-H, Kumar S, Chen P-J, Wu Y-H, Lai C-L, Hsieh H-L, Sung P-J, Hwang T-L (2020). Mar Drugs.

[15] Sica D, Musumeci D (2004). Steroids.

[16] Adinolfi R, Migliuolo A, Piccialli V, Sica D (1994). J Nat Prod.

[17] Jäälaid R, Järving I, Pehk T, Lille Ü (1998). Proc Est Acad Sci, Chem.

[18] Kuhl A, Kreiser W (1998). Tetrahedron Lett.

[19] Jäälaid R, Järving I, Pehk T, Parve O, Lille Ü (2001). Nat Prod Lett.

[20] Kongkathip B, Hasakunpaisarn A, Boonananwong S, Kongkathip N (2010). Steroids.

[21] Salvador J A R, Silvestre S M, Moreira V M (2012). Curr Org Chem.

[22] Warnke M, Jung T, Dermer J, Hipp K, Jehmlich N, von Bergen M, Ferlaino S, Fries A, Müller M, Boll M (2016). Angew Chem, Int Ed.

[23] Ferrandi E E, Bertuletti S, Monti D, Riva S (2020). Eur J Org Chem.

[24] Li A, Acevedo-Rocha C G, D’Amore L, Chen J, Peng Y, Garcia-Borràs M, Gao C, Zhu J, Rickerby H, Osuna S (2020). Angew Chem, Int Ed.

[25] Acevedo-Rocha C G, Gamble C G, Lonsdale R, Li A, Nett N, Hoebenreich S, Lingnau J B, Wirtz C, Fares C, Hinrichs H (2018). ACS Catal.

[26] Kerr R G, Rodriguez L C, Keliman J (1996). Tetrahedron Lett.

[27] Wüst F, Carlson K E, Katzenellenbogen J A (2003). Steroids.

[28] Marwah P, Marwah A, Lardy H A (2003). Tetrahedron.

[29] Petrusma M, Dijkhuizen L, van der Geize R (2009). Appl Environ Microbiol.

[30] Petrusma M, Hessels G, Dijkhuizen L, van der Geize R (2011). J Bacteriol.

[31] Kirihara M, Osugi R, Saito K, Adachi K, Yamazaki K, Matsushima R, Kimura Y (2019). J Org Chem.

[32] Quan J, Tian J (2011). Nat Protoc.

