# Supplementary material for: Designed whole-cell-catalysis-assisted synthesis of 9,11-secosterols
Source: Beilstein J Org Chem. 2021 Mar 1;17:581–8. doi: 10.3762/bjoc.17.52 (PMC7940815; doi:10.3762/bjoc.17.52)

BEILSTEIN JOURNAL OF ORGANIC CHEMISTRY

Supporting Information

for

Designed whole-cell-catalysis-assisted synthesis of 9,11-secosterols

Marek Kõllo, Marje Kasari, Villu Kasari, Tõnis Pehk, Ivar Järving, Margus Lopp, Arvi Jõers and Tõnis Kanger

Beilstein J. Org. Chem. **2021,** *17,* 581–588. doi:10.3762/bjoc.17.52

General material and methods for the construction of the biocatalyst as well as NMR spectra of synthesized compounds

License and Terms: This is a supporting information file under the terms of the Creative Commons Attribution License (https://creativecommons.org/licenses/by/4.0). Please note that the reuse, redistribution and reproduction in particular requires that the author(s) and source are credited and that individual graphics may be subject to special legal provisions.
The license is subject to the *Beilstein Journal of Organic Chemistry* terms and conditions: (https://www.beilstein-journals.org/bjoc/terms)

Table of contents

Supporting material and methods S2
Figure S1: Expression analysis of KSH proteins S3
Figure S2: Map of kshA5 and kshB expression vector pAJ30 S4
NMR spectra S5

Supporting material and methods

Polyacrylamide gel electrophoresis (SDS-PAGE)
E. coli BL21 (DE3) cells carrying pAJ30 plasmid for expression of *kshA5* and *kshB* were pregrown overnight at 37 °C. Next morning, 200 mL of LB medium in a 2 L baffled flask was inoculated with 1 mL of a preculture. The cultures were grown until OD600 = 0.1 at 37 °C with continuous shaking at 220 rpm, and then the expression was induced by adding 1 mM IPTG. Thereafter, the temperature of the cultures was decreased to 30 °C, and the growth was continued with continuous shaking at 220 rpm overnight. 1 mL of culture samples were collected prior to induction of kshA5 and kshB synthesis and 2, 4 and 20 hours postinduction. The cells from these 1 mL samples were harvested by centrifugation at 11,000 × g and resuspended in 1x SDS sample buffer to final concentration of 5 OD600 units/mL (cells from 1 mL of culture at optical density OD600 = 1 is defined as 1 OD600 units/mL of sample), followed by heating the samples at 97 °C for 10 minutes to lyse the cells and denature the proteins. Either 0.03 or 0.003 OD600 units of each sample was loaded to a 12% polyacrylamide gel for protein staining or western blotting, respectively, and separated by gel electrophoresis. The proteins in the gel were stained using InstsantBlue Coomassie Protein Stain (Abcam, ab119211).

Western blotting
Protein lysates separated by SDS-PAGE were transferred to a PVDF membrane (Trans-Blot Turbo Mini 0.2 µm PVDF Transfer Packs, BioRad #1704156) using Trans-Blot Turbo Transfer System (BioRad) according to recommended protocol by the manufacture. HisProbe™-HRP Conjugate (ThermoFisherScientific, 15165) and SuperSignal™ West Pico PLUS Chemiluminescent Substrate (ThermoFisherScientific, 34579) was used to illuminate 6His-tagged KSH proteins and recorded in LI-COR Fc imaging system.

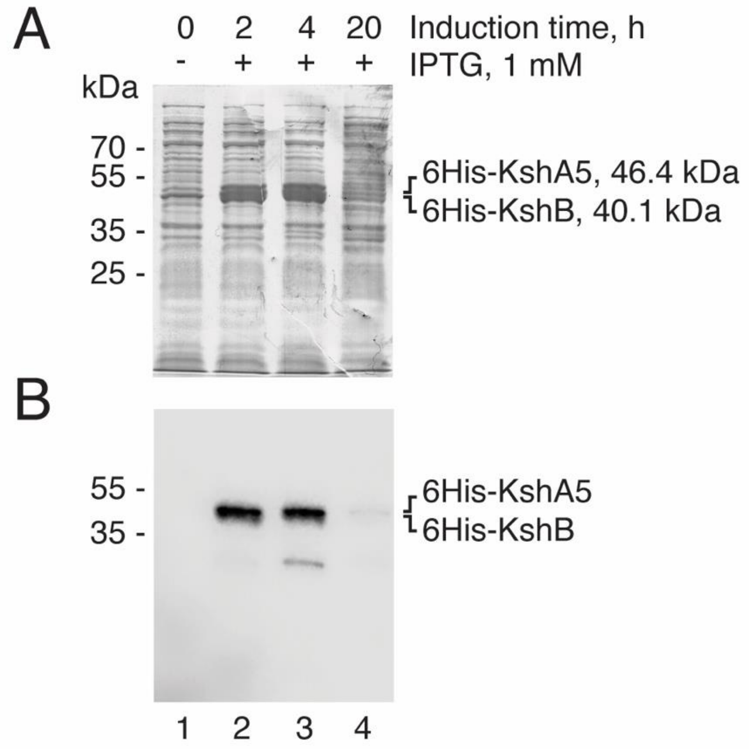

Figure S1: Expression analysis of KSH proteins. The expression of 6His-tagged kshA5 and kshB was monitored in *E. coli* BL21 (DE3) cells carrying pAJ30 plasmid. The culture was grown in LB medium and at OD600 = 0.1. Protein synthesis was induced with 1 mM IPTG (final concentration). 1 mL samples were collected at indicated time points after induction of the protein synthesis and subjected to SDS polyacrylamide gel electrophoresis (A) and western blotting (B).

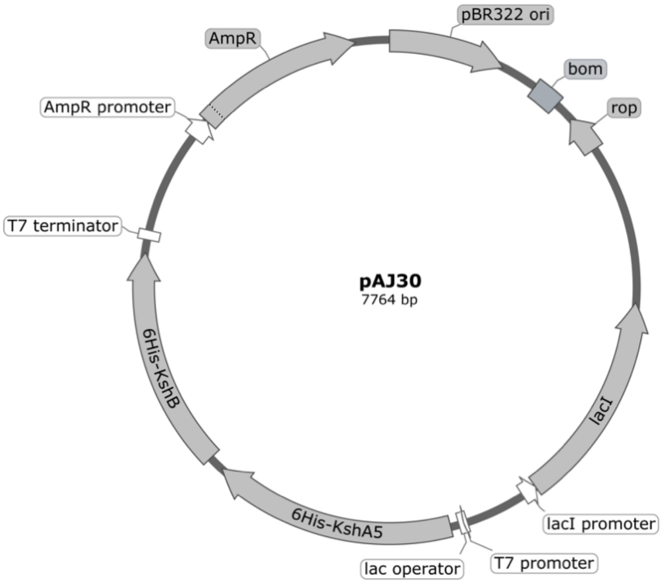

Figure S2: Map of kshA5 and kshB expression vector pAJ30. Codon-optimized *kshA5* and *kshB* with N-terminal 6His-tags were placed under the control of T7 promoter as a single operon in pET21a plasmid. The map was created using SnapGene software. The sequence of the plasmid is available as GenBank file (Supplementary File 2).

NMR spectra

^{1}H NMR spectrum of **2**

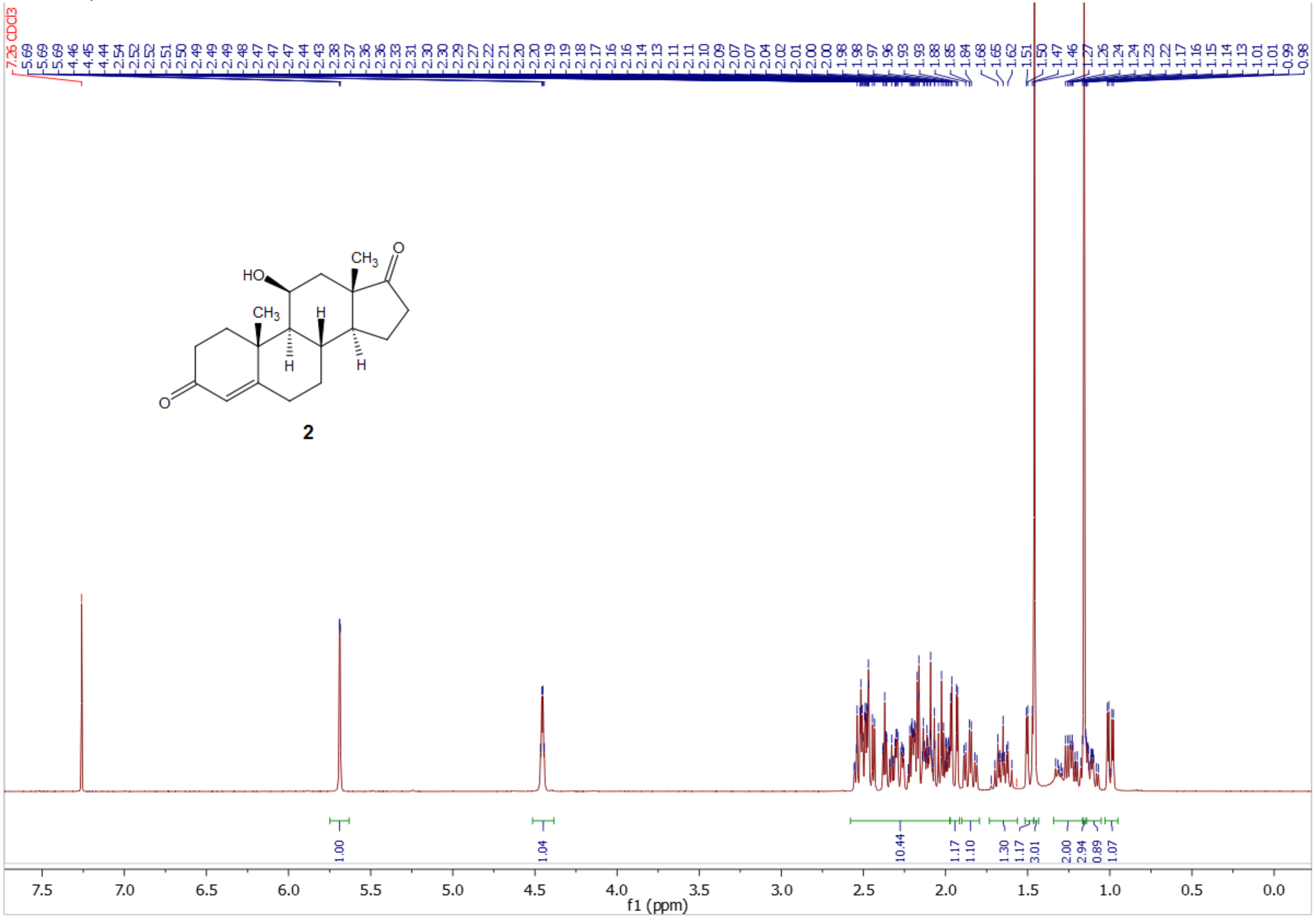

^{13}C NMR spectrum of **2**

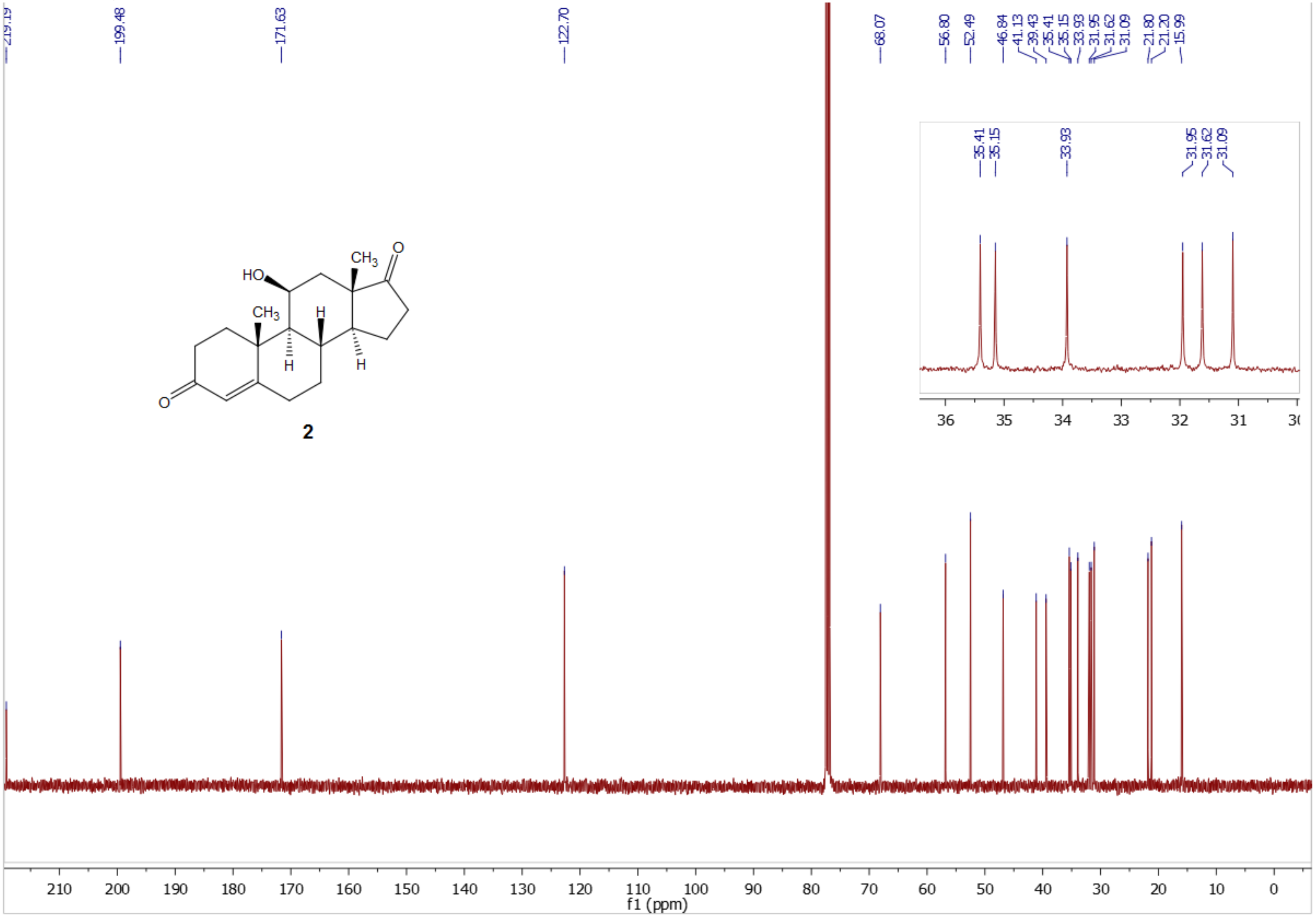

^{1}H NMR spectrum of **3**

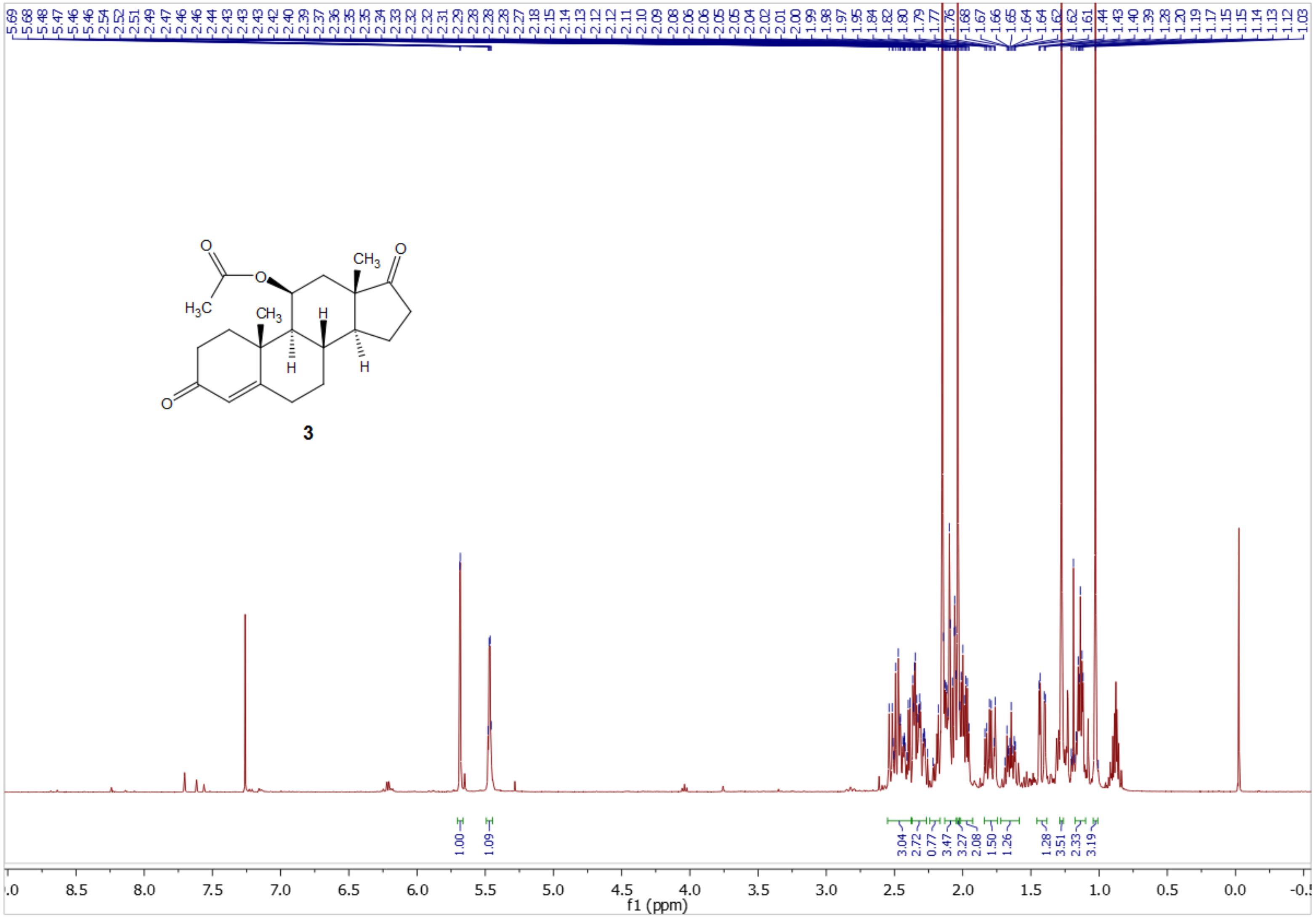

^{13}C NMR spectrum of **3**

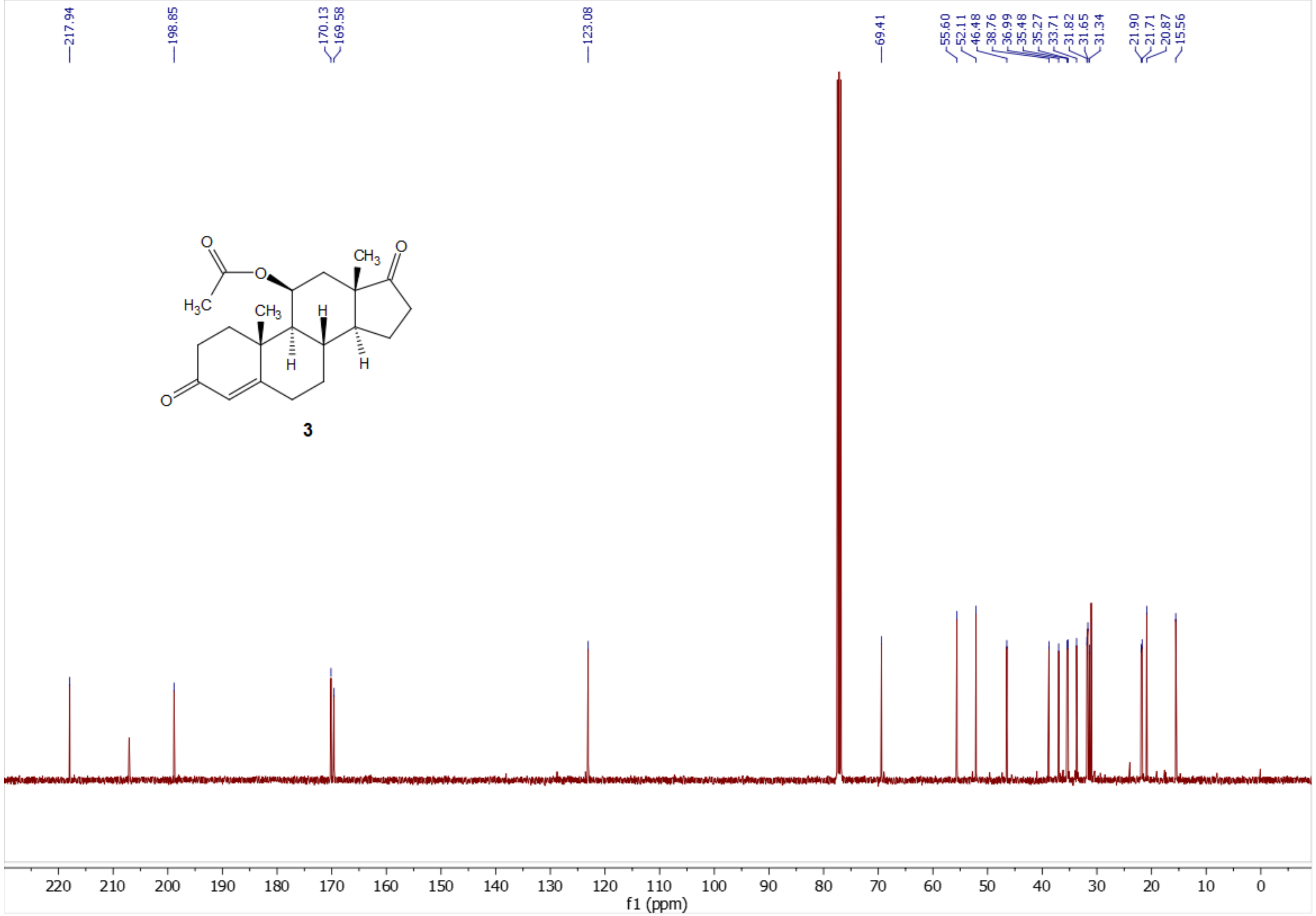

^{1}H NMR spectrum of **4**

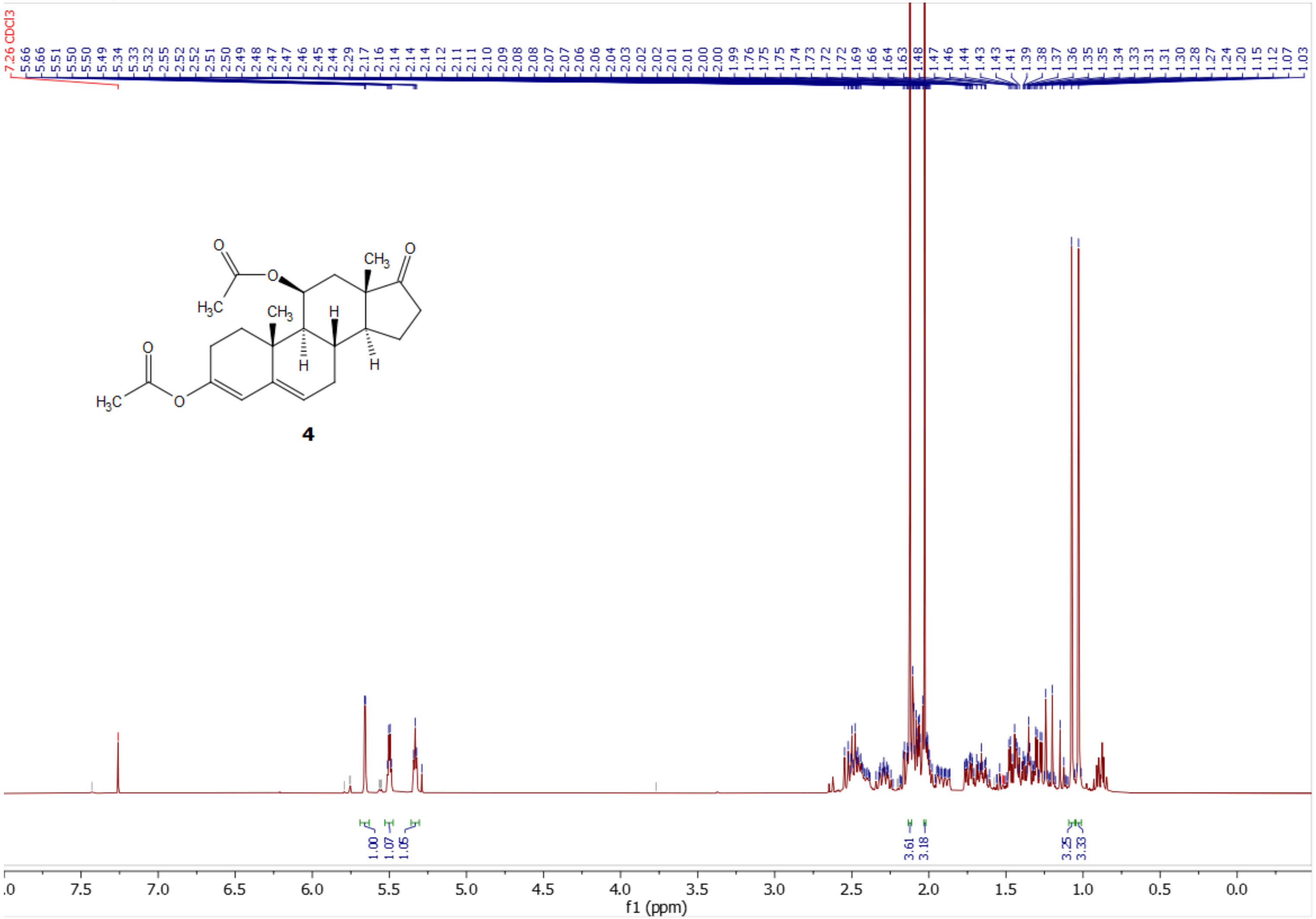

^{13}C NMR spectrum of **4**

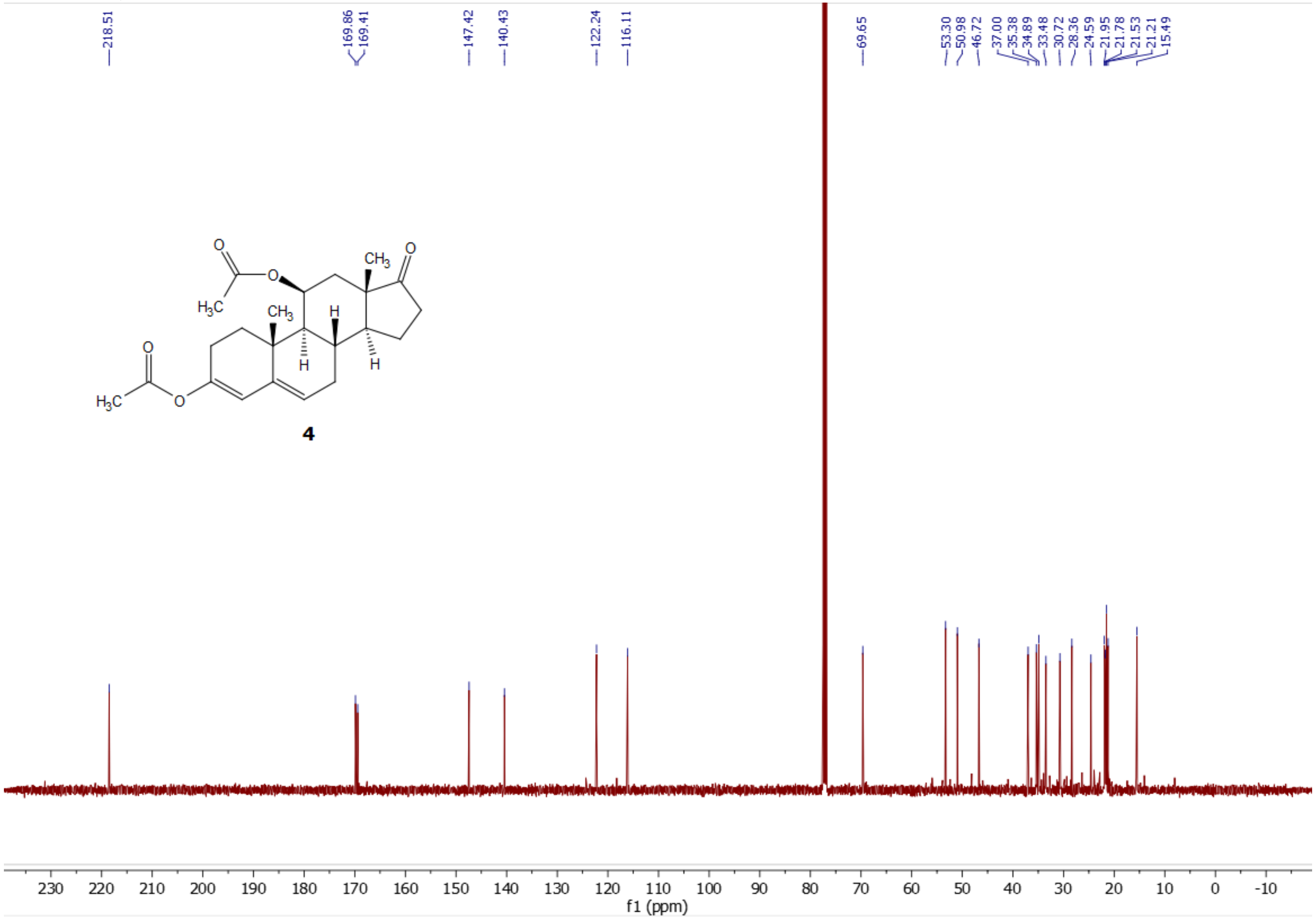

^{1}H NMR spectrum of **5**

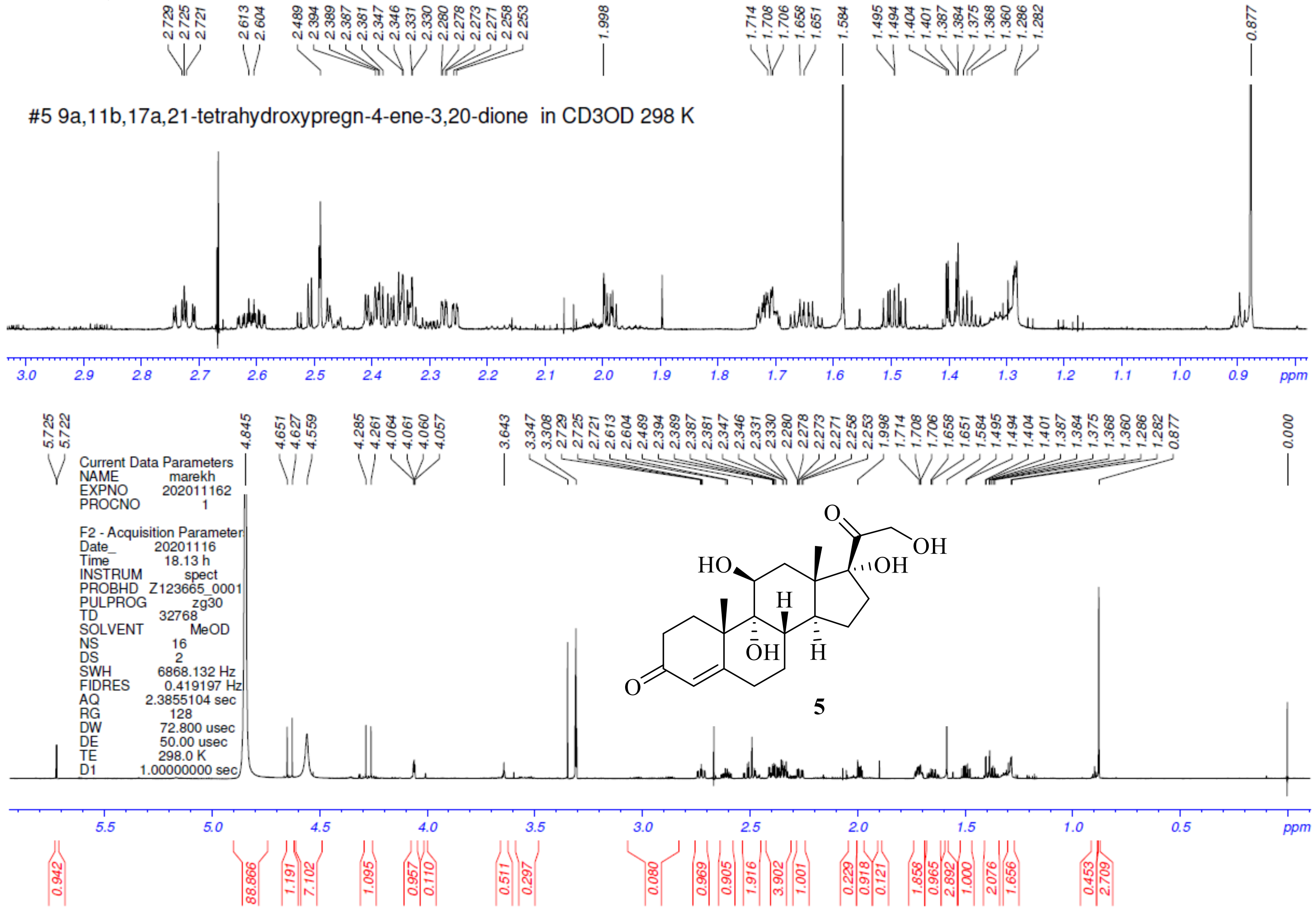

^{13}C NMR spectrum of **5**

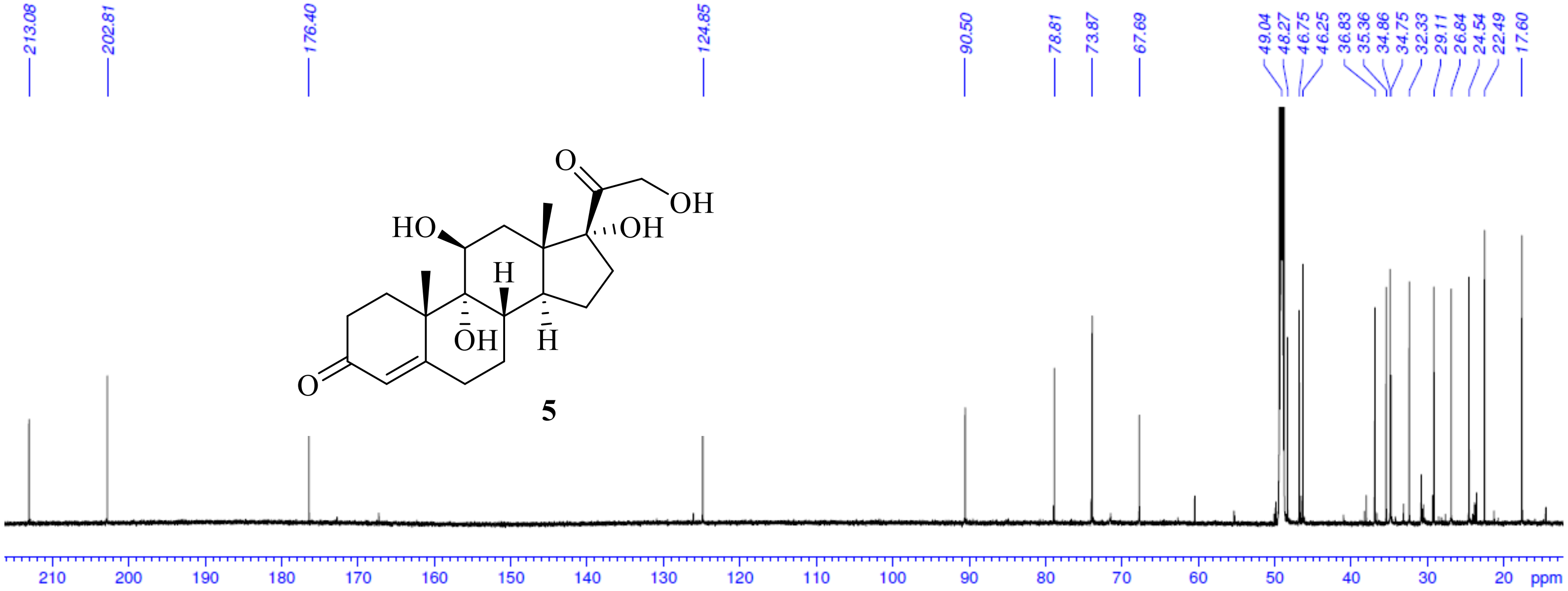

Current Data Parameters
NAME marekc
EXPNO 202011160
PROCNO 1

F2 - Acquisition Parameters
Date_ 20201116
Time 18.10 h
INSTRUM spect
PROBHD Z123665_0001 (
PULPROG zgpg30
TD 131072
SOLVENT MeOD
NS 840
DS 16
SWH 44642.855 Hz
FIDRES 0.681196 Hz
AQ 1.4680064 sec
RG 1290
DW 11.200 usec
DE 19.15 usec
TE 298.0 K
D1 1.00000000 sec

#5 9a,11b,17a,21-tetrahydroxypregn-4-ene-3,20-dione in CD3OD 298 K

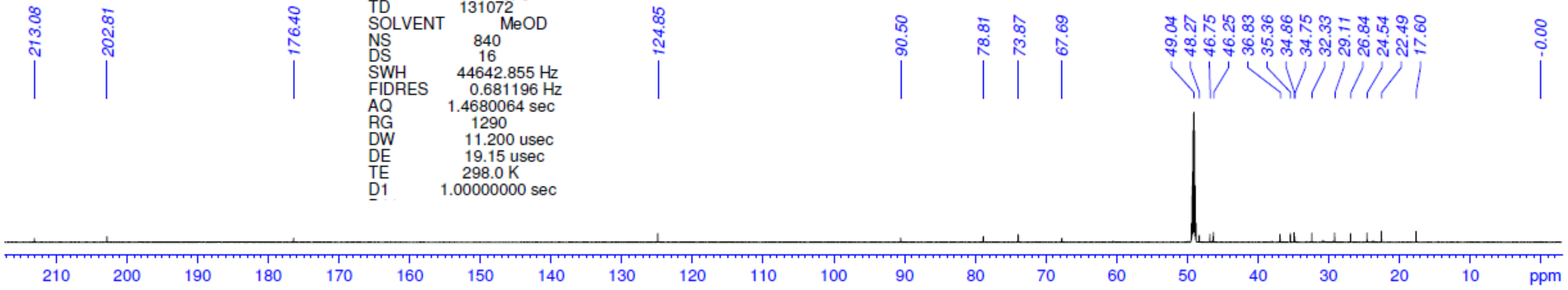

^{1}H NMR spectrum of **6**

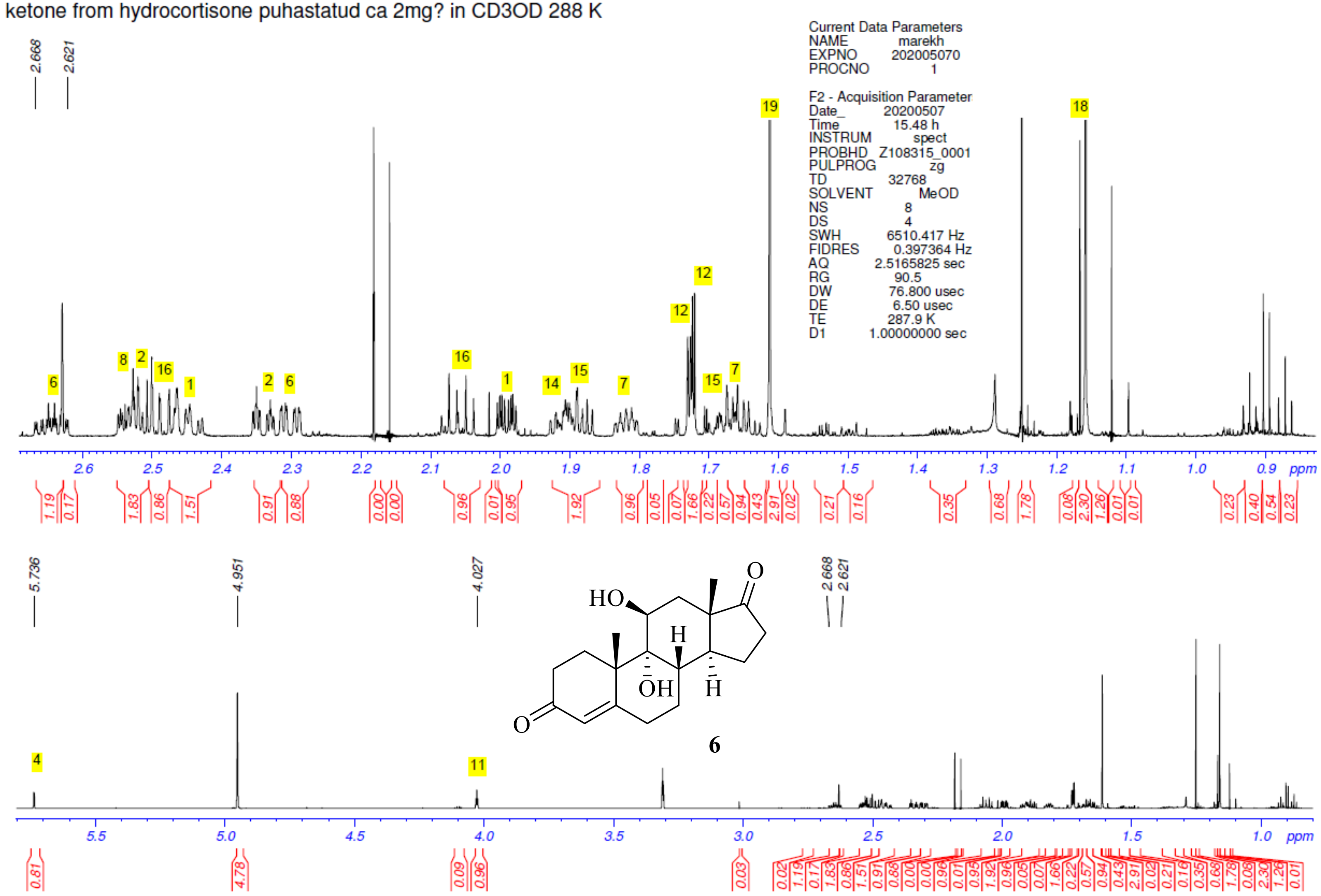

6

^{13}C NMR spectrum of **6**

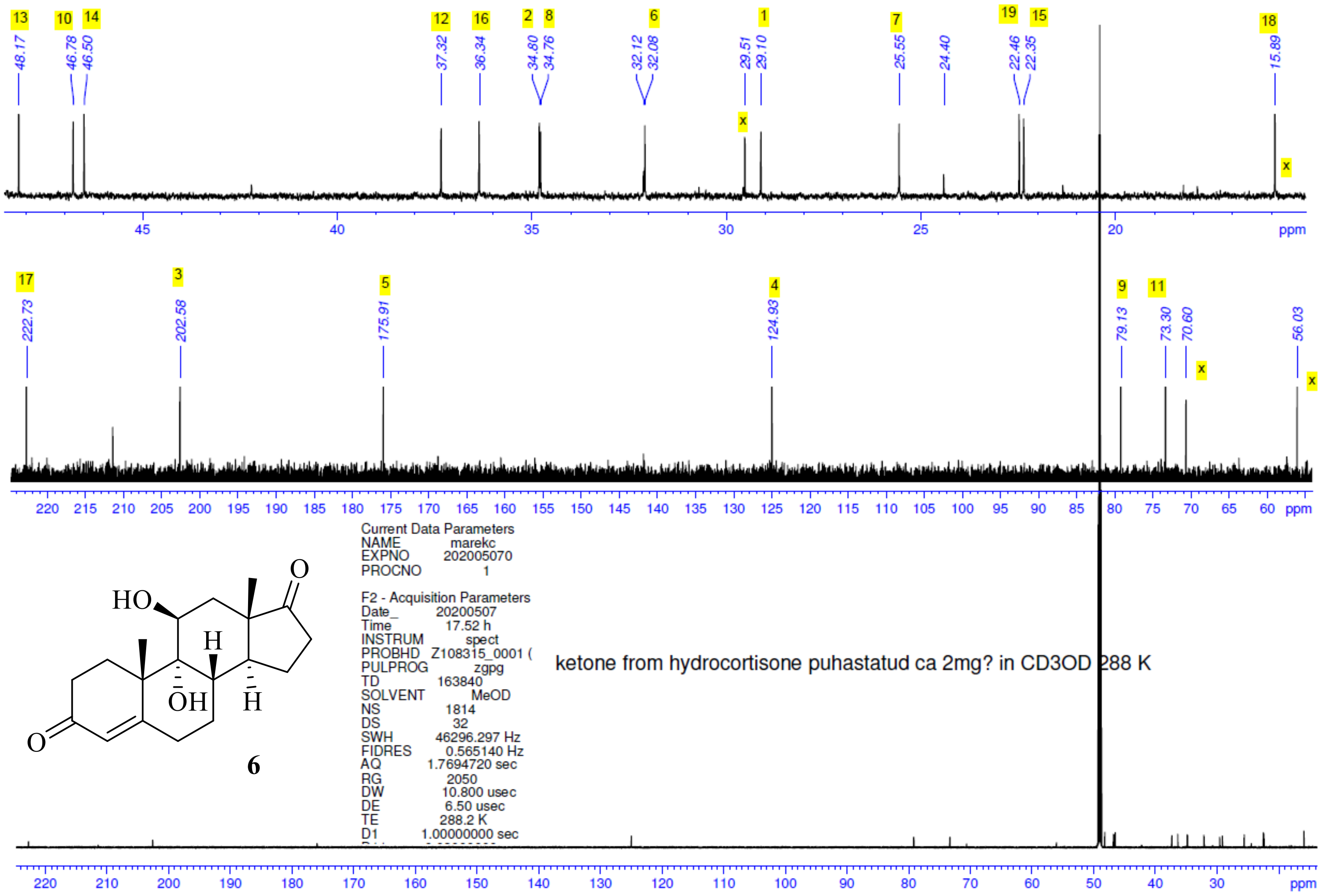

^{1}H NMR spectrum of **7**

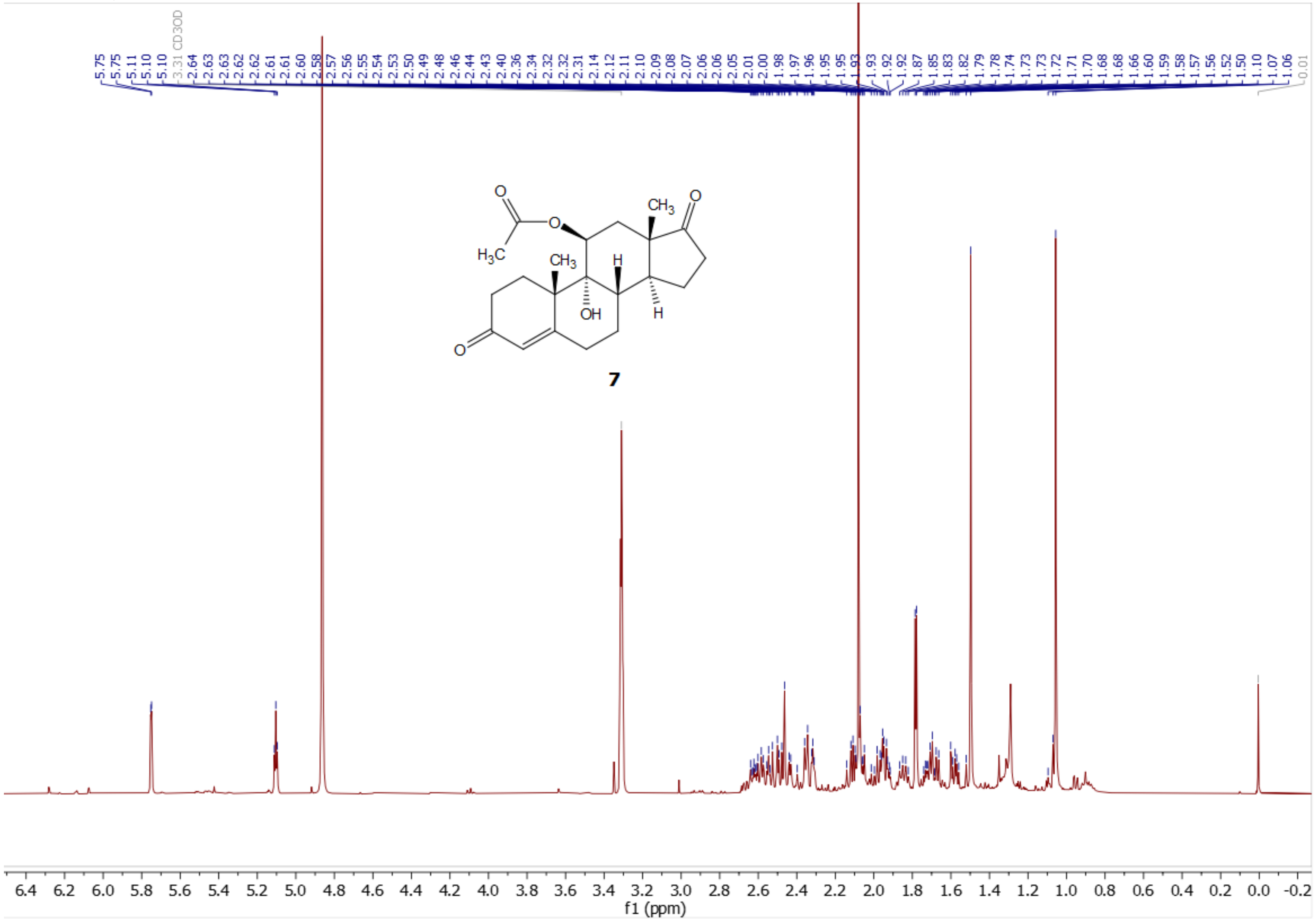

^{13}C NMR spectrum of **7**

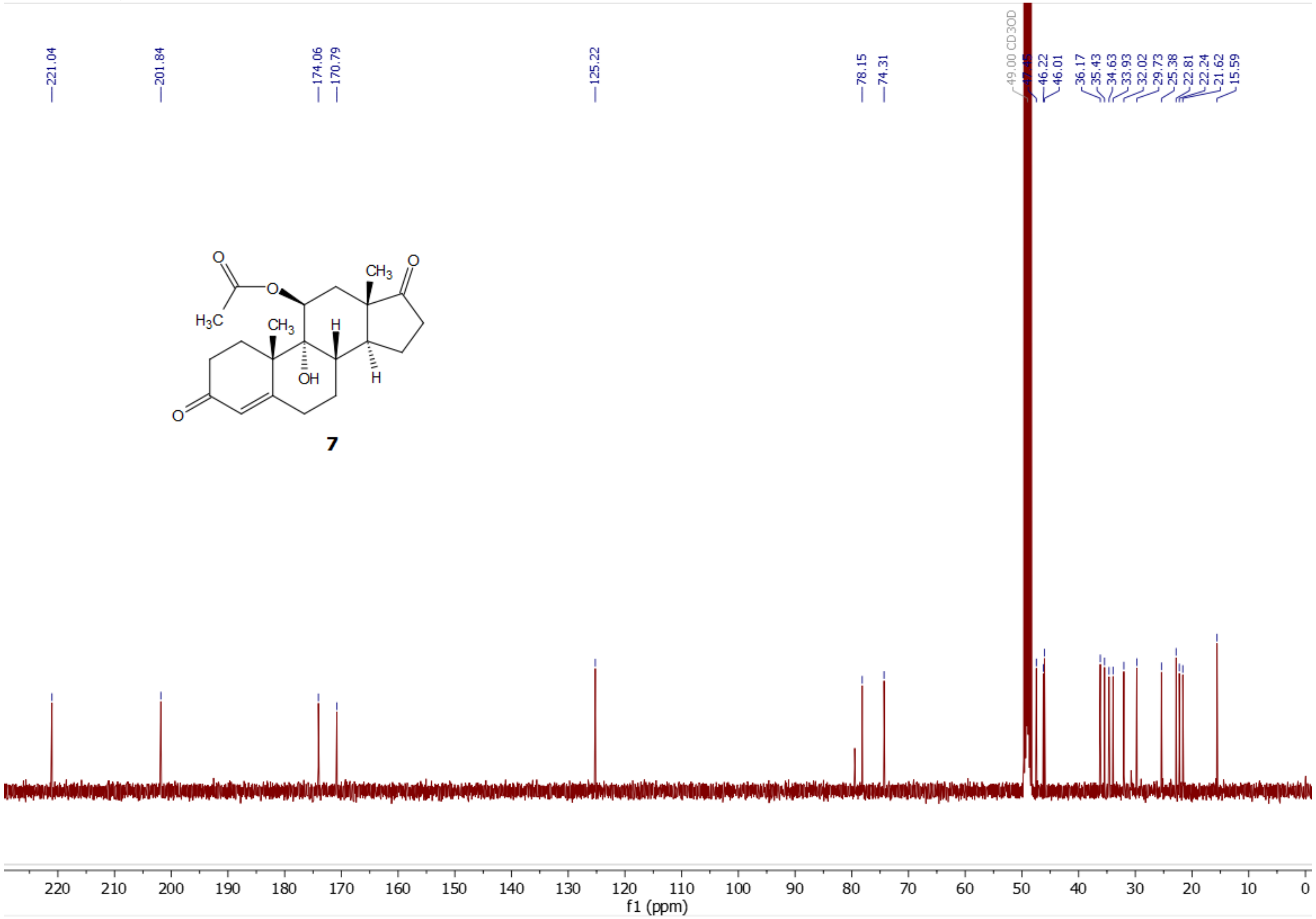

^{1}H NMR spectrum of **8**

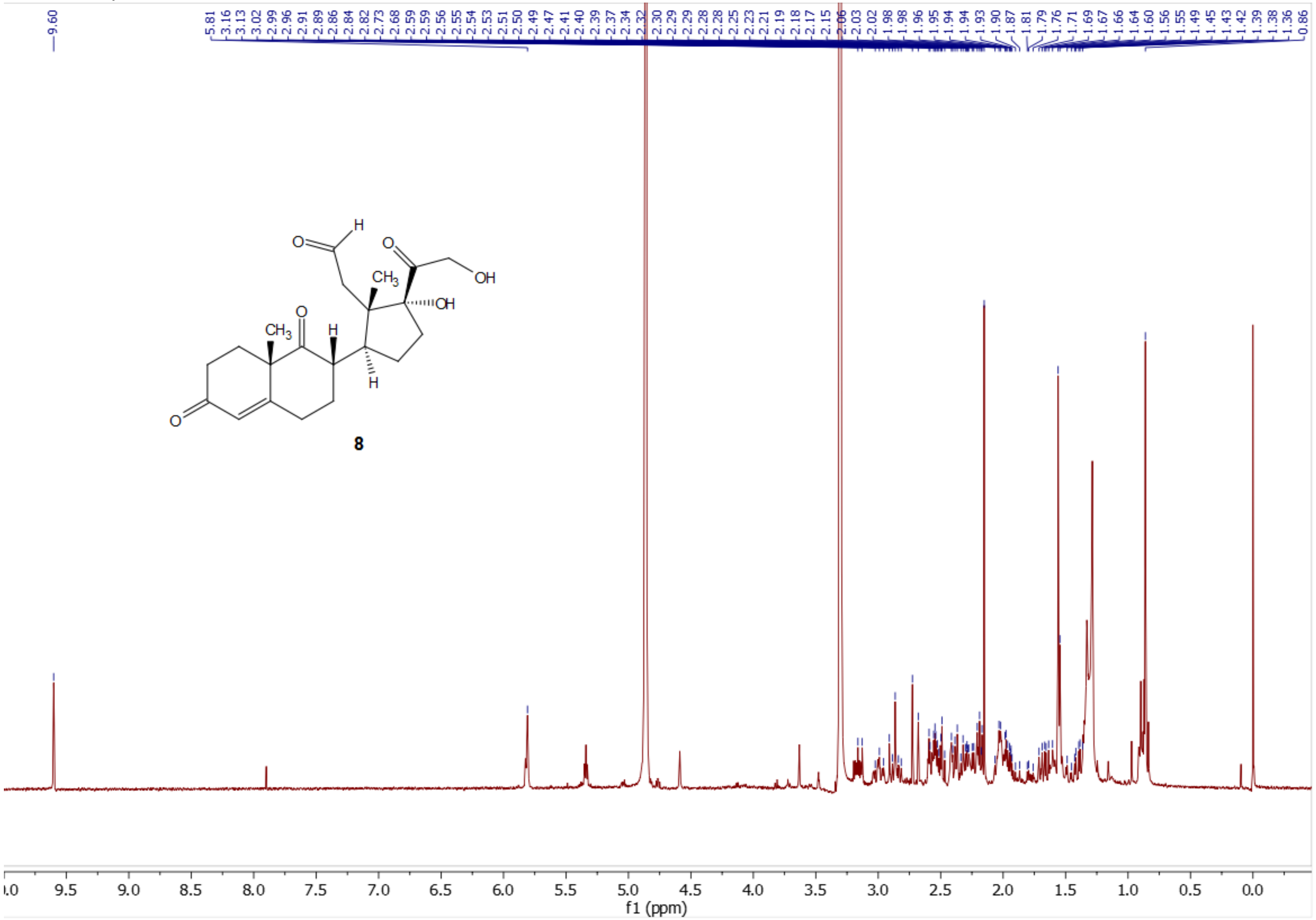

^{13}C NMR spectrum of **8**

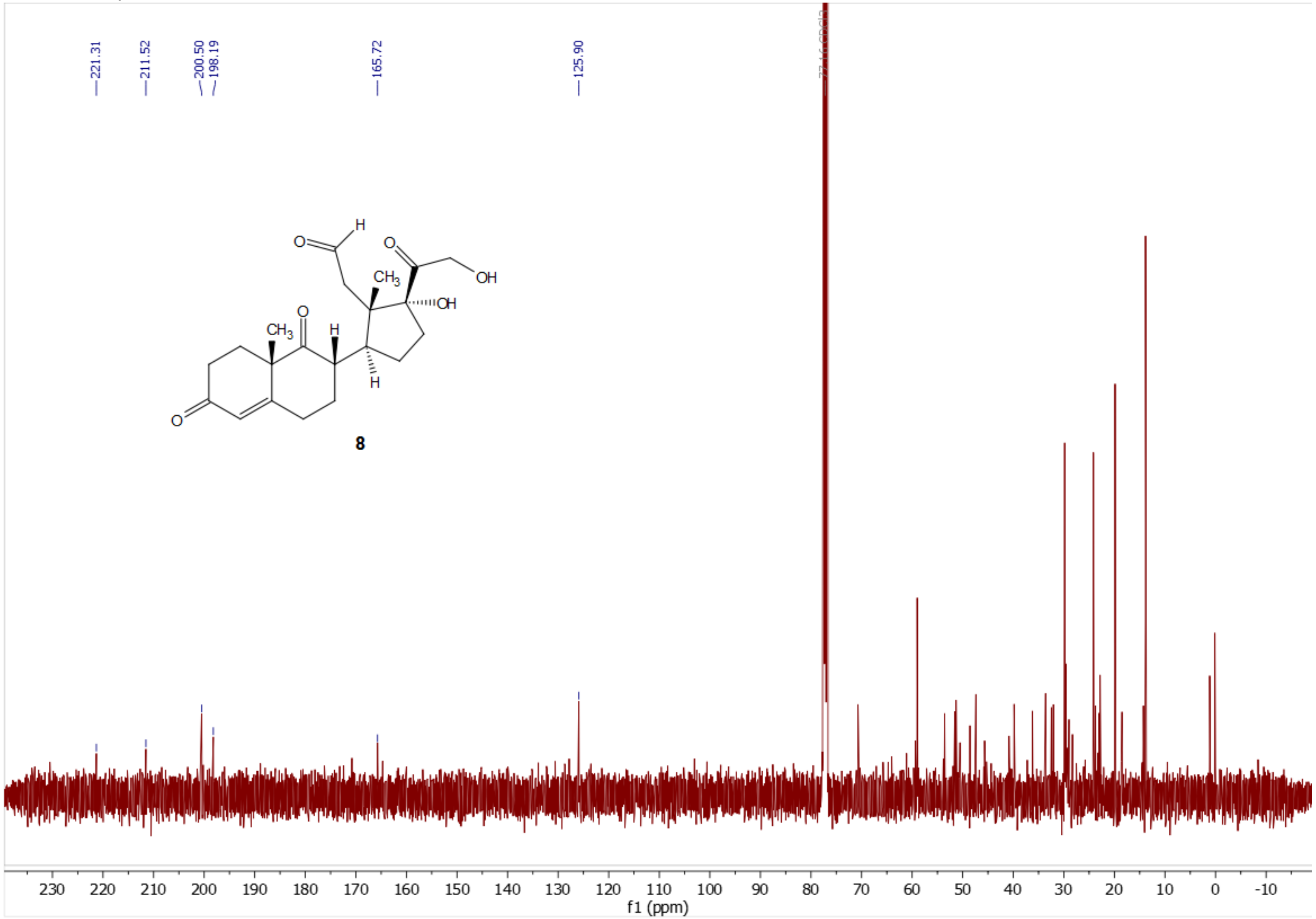

^{1}H NMR spectrum of **9**

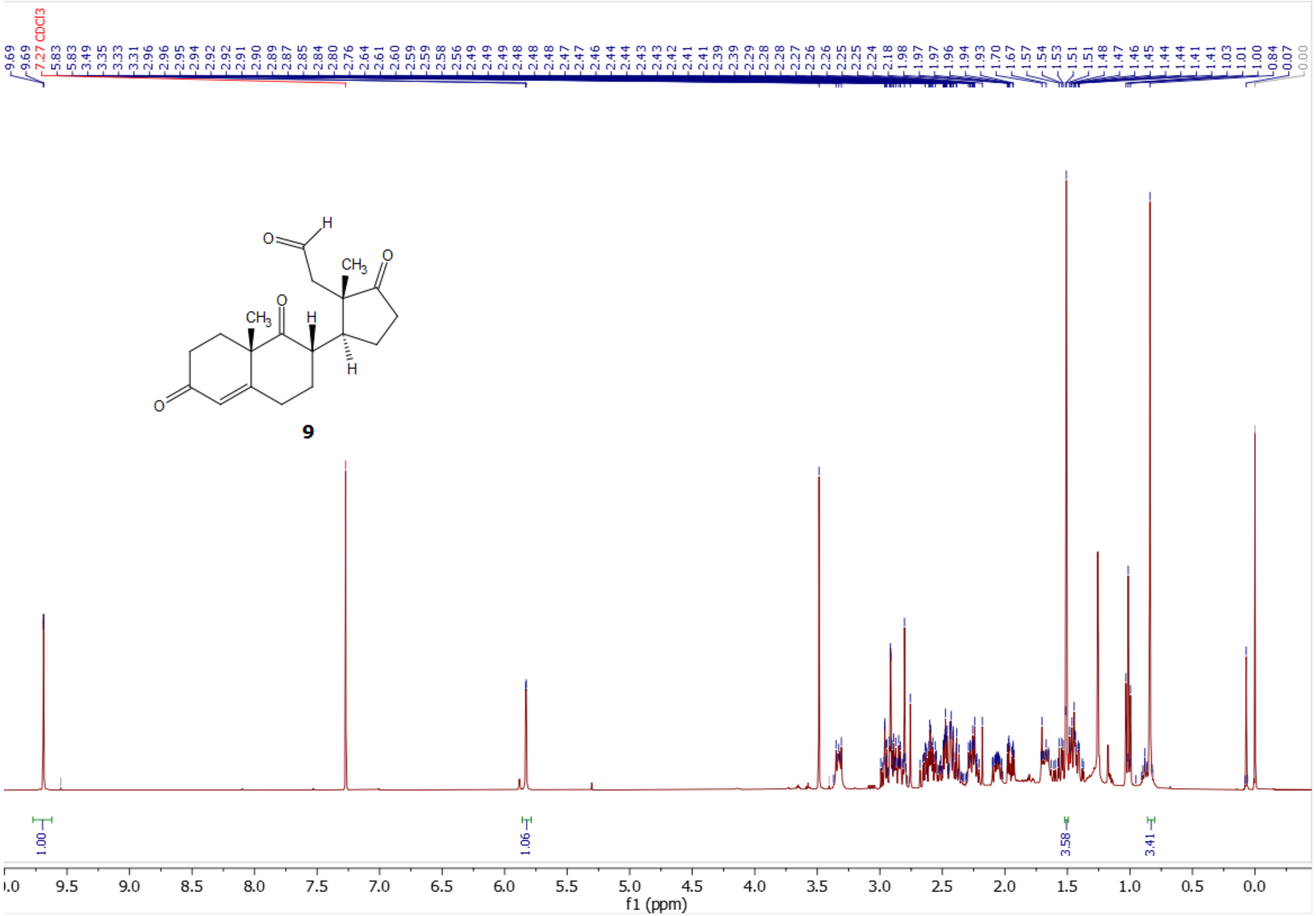

^{13}C NMR spectrum of **9**

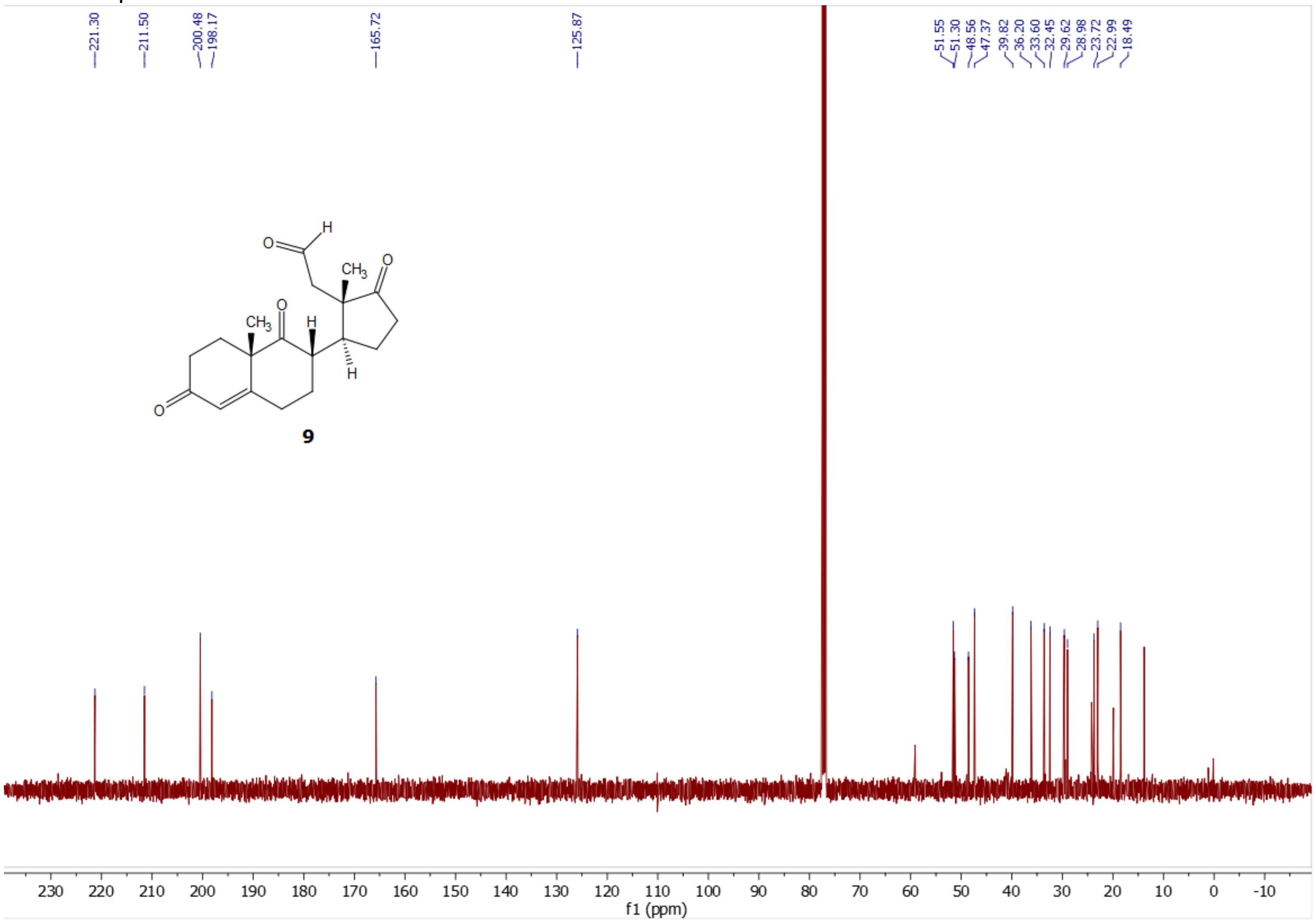

Supplement: File 1 — General material and methods for the construction of the biocatalyst as well as NMR spectra of synthesized compounds. [file Beilstein_J_Org_Chem-17-581-s001.pdf]
